# A New Year's Wish List for Authors, Reviewers, Readers—and Ourselves

**DOI:** 10.1371/journal.pmed.1000203

**Published:** 2009-12-22

**Authors:** The PLoS Medicine Editors

## Abstract

The *PLoS Medicine* editors offer some suggestions for New Year's resolutions for authors, reviewers, editors, and readers to consider.

For the most part, publishing of research is a gratifying experience for journals and authors. Such publishing is predicated, above all, on trust. Authors need to trust that a journal's reviewers and editors provide a fair review process of their papers. And of course journals need to trust authors to provide a fair, honest, and complete account of their work. Only then can readers have trust in the articles that are published.

Looking back over the past year at PLoS, as well as across the broader landscape of academic publishing, it would be hard to conclude that this trusting relationship has not been shaken rather profoundly at times. Editors have sometimes been taken unawares by ghost and guest authors, manipulation of figures, lack of authors' willingness to share data, failure to register trials, and salami-slicing of data to produce the “least publishable unit.” From an author's perspective—reflected in our survey of authors earlier this year, for example—the relationship may not have been as rosy as it should have been. Long decision times, hypercritical reviewers, seemingly impossible demands at the production stage, and rejection after an extended review process are always hard for authors to bear.

So in the spirit of a soon-to-be New Year we'd like to gently offer some suggestions for resolutions for all authors, reviewers, readers, and editors to ponder. We hope that no one will take offence and trust that our choices will become clear as we explain.

## For authors…

- *My authorship list will be complete and accurate, and everyone named will deserve authorship; in particular there will be no ghost or guest authors*.

In case anyone doubts that such a resolution is necessary, look at the furor in July 2009 about the involvement of ghost authors in Wyeth's marketing of Prempro that came to light after our intervention, with *The New York Times*, in a court case [1]. Closer to home, a study presented at the Sixth International Congress on Peer Review in September 2009 suggested that we at *PLoS Medicine* have both guest and ghost authors lurking in our papers [2].

- *I'll have all the data to back up my paper and its images, and none of it will have been manipulated to look prettier than it really was when it came off the scanner/computer.*

Unfortunately, many authors are unable to provide the original data underpinning their studies and images. For example, a preliminary survey by *PLoS Biology* and *PLoS Medicine* in 2009 found that 15% of all authors who have been asked for original data could not find it. We don't yet know how often, say, a misplaced gel means manipulation of data, but being fooled even once would be too often, so we will continue to routinely check all images on accepted papers.

- *I'll stand by my agreement to share all the data that underpins my research* [*3*] *. As part of that commitment I will share the study protocol for that research, when requested by editors*.

At *PLoS Medicine*, we consider the minimization of reporting biases an important part of our role, and duly require authors submitting reports of clinical trials to also provide a copy of the prespecified study protocol. We have on occasion had our requests for protocols met with the response “How would you like the document written?” Can it surprise anyone that such a response shakes our confidence in the prespecified nature of the analyses?

- *I'll make sure I've complied with all the requirements for trial registration.*

Trials are still submitted to us that have not been registered. Compulsory trial registration has been in place for more than three years now, and it is clearly laid out in our author guidelines [4] We therefore will reject unregistered trial reports. And while we're on the subject of registration, why not register all human subjects research? An increasing number of researchers are doing so in ClinicalTrials.gov, and there are compelling reasons for expanding such registration.

## For reviewers…

- *If I'm unable to review a paper, I'll let the editor know within a few days of receiving the request to review.*

Editors expect reviewers to say “no” to requests quite often—we understand you are busy. But if you can let us know quickly, we can then provide authors with a faster editorial peer review process. We also expect reviewers to declare competing interests related to the paper or the authors [5].

- *If I promise to review I will do so.*

Late reviews cause even more problems than late responses to review requests. Not only does failure to provide a timely review set the evaluation process back, but reviews that never materialize also raise questions about a reviewer's motives for agreeing to review in the first place.

- *I'll be polite when I review the paper.*

The tone of a review can mar an otherwise very helpful review. Reviews also aren't the place to impugn the authors' motives, unless of course you have incontrovertible evidence. Even so, it is best to stick to the facts. We'll take it very seriously if you contact us to say that there is something we must scrutinize further.

## For our readers…

- *Will you resolve to talk to us?*

We'd urge our readers to tell us more about what you want from the journal and its online functionality, and what you think of the articles themselves. Read, rate, and reuse the papers. Open access journals are only partially fulfilling their mission if the papers they publish remain embedded in the journals they were originally published in and not reused or redistributed.

## And, finally, for editors…

We're sure our authors will have opinions on this but here's what we resolve to do.

- *We'll be quick, efficient, and courteous in our process.*

We understand how important your work is. We have all been authors too. Presubmission inquiries have been successful in reducing our first decision time to about 24 hours. We are now actively looking at all aspects of our process to decrease time to decisions later in the process.

- *We'll review the composition of our editorial and reviewer boards* in an attempt to be inclusive in the people we get to advise us.
- *We'll listen to our authors and reviewers if they have complaints or suggestions.*

So please, if we send you a survey, fill it in. We love feedback, even if it's not positive.

- *We'll continue to advocate for the highest level of publishing ethics*.

We will continue to publish editors' competing interests [6] and abide by the editors' code of conduct developed by the Committee on Publication Ethics (COPE) [7].

- *We'll continue to innovate*.

We recognize there is still a ways to go before open access journals fulfill the promise of a truly integrated information resource. So far this year we have instituted new ways of publishing by using our new TOPAZ software program, which provides enhanced ability to comment on articles. The recently launched article level metrics on all the PLoS journals [8] now provide new ways of assessing the true impact of papers. But we recognize the need to do more.

Here's one more resolution we'd like to share with everyone. By the time this editorial is published, leaders representing signatories to the United Nations Framework Convention on Climate Change will have met in Copenhagen. Whatever the outcome of the summit, the solution to climate change can't just rest with politicians – it is the responsibility of us all. We've just signed up to 10:10 [9] (we commit to cutting 10% of our carbon emissions in 2010) and we'd encourage everyone to do the same. In addition to being an important practical action, it also shows a collective will toward constructive change, something that our authors, reviewers, and readers have already shown in their support of open access. We'll be writing more about this in 2010. Between now and then have a very a happy and peaceful New Year, and if you can find time between the celebrations, write and tell us your resolutions.
